# Patient satisfaction with doctor-patient interactions: a mixed methods study among diabetes mellitus patients in Pakistan

**DOI:** 10.1186/s12913-017-2094-6

**Published:** 2017-02-21

**Authors:** Aisha Jalil, Rubeena Zakar, Muhammad Zakria Zakar, Florian Fischer

**Affiliations:** 1 Department of Sociology, Institute of Social and Cultural Studies, University of the Punjab, Lahore, Pakistan; 2 Department of Public Health, Institute of Social and Cultural Studies, University of the Punjab, Lahore, Pakistan; 3 Institute of Social and Cultural Studies, University of the Punjab, Lahore, Pakistan; 4 Department of Public Health Medicine, School of Public Health, Bielefeld University, Bielefeld, Germany

**Keywords:** Diabetes, Pakistan, Patient satisfaction, Doctor-patient interaction

## Abstract

**Background:**

Patient satisfaction with doctor-patient interactions is an indicator of physicians’ competence. The satisfaction of diabetes patients is rarely studied in public diabetes clinics of Pakistan. Thus, this study aims to analyse the association between patient satisfaction and five dimensions of medical interaction: technical expertise, interpersonal aspects, communication, consultation time, and access/availability.

**Methods:**

A cross-sectional mixed methods study was conducted during July and August 2015 in the largest public diabetes outpatient clinic in Punjab province. We used the criterion sampling method to identify 1164 patients who: (i) were adult (18 years and above), (ii) had diabetes mellitus, (iii) had made at least three previous visits to the same clinic. The data was collected through face-to-face interviews. The structured part of the questionnaire was based on demographic characteristics and the Patient Satisfaction Questionnaire (PSQ-III). We translated the questionnaire into Urdu and pretested it with 25 patients in a similar context. Data storage and analysis were carried out using SPSS (version 22.0). Bivariate analyses and multinomial logistic regression model were used to generate the quantitative findings. Out of the 1164 eligible patients approached for interviews, 1095 patients completed the structured questionnaire and 186 respondents provided qualitative information in comments section. We conducted a thematic content analysis of qualitative responses in order to explain the quantitative findings.

**Results:**

Demographic characteristics such as gender, education and occupation were significantly associated with the levels of patient satisfaction. The dimensions of doctor-patient interaction were significantly associated with patient satisfaction: technical expertise (OR = .87; 95% CI = .84–.91), interpersonal aspects (OR = .82; 95% CI = .77–.87), communication (OR = .83; 95% CI = .78–.89), time dimension (OR = .90; 95% CI = .81–.99) and access/availability (OR = .78; 95% CI = .72–.84). Several factors involving doctors’ incompetence, such as inappropriate handling of critical cases, inaccurate diagnose, excessive reliance on medical tests, absence of physical examination, non-availability of specialist doctors, and experimentation by trainee doctors were related to patient dissatisfaction.

**Conclusion:**

The findings of this study highlight a need to develop the interpersonal and clinical skills of doctors in order to improve the quality of doctor-patient interactions in public clinics for diabetes in Pakistan. Prospective researches should explore context-specific factors that form patient satisfaction.

**Electronic supplementary material:**

The online version of this article (doi:10.1186/s12913-017-2094-6) contains supplementary material, which is available to authorized users.

## Background

Health service researchers approve patient satisfaction as the key outcome indicator of medical care quality. Patient satisfaction with the doctor-patient interaction indicates the level of doctor’s success and competence in service provision [1]. Maintaining good technical as well as interpersonal skills is essential for the doctors to satisfy their patients [2]. In addition, the demonstration of professionalism and ethical practice are also required to meet the expectations of patients [3]. The technical expertise of physicians is regarded as consisting of: maintaining an appropriate level of experience, ability to diagnose, performance of clinical procedures, prescribing medicine and learning about the latest medical developments [4]. Moreover, the success of technical procedures, treatment and medication depends upon favourable communication with patients.

Patient’s consultation experience is positively associated with patient’s decision to re-visit a doctor [5]. Empirical literature reveals that dissatisfied patients are more likely to discontinue seeking consultation with a physician whom they perceive as incompetent [6, 7]. Likewise, the delays in seeking medical consultation and self-medication are also frequently observed among dissatisfied patients [2]. In scarce resource settings where alternatives are unavailable for patients from lower socio-economic background, patients continue seeking consultation in the same clinic irrespective of dissatisfaction.

Unlike developed countries, the doctors are not made to comprehend the importance of ethical practice and communication skills during medical training in Pakistan [2]. Physicians working in public hospitals deal with patients of a lower socio-economic class with negligible health awareness and poor hygiene [4, 8]. Understanding the patients and making them understand is the big challenge with which physicians are confronted in public outpatient clinics in Pakistan [9]. According to a Gallop Survey, Pakistan scored the lowest in world on the Global Doctor-Patient Communication Assessment test in year 2011 with five points in contrast to the highest score 66 points was recorded for Ireland [10].

Pakistan is one of the world’s largest countries with highest prevalence of diabetes mellitus (6.7% raw national prevalence), which is expected to rise further in the years to come [11]. In order to rationalise the process of planning for diabetes prevention and control, the timely evaluation of diabetes care facilities is a prerequisite [12]. Unfortunately, the government is not targeting reforms in public diabetes care facilities for patients seeking free-of-cost consultation and treatment. According to the World Health Organisation, public health expenditure in Pakistan is also amongst the lowest in the world, from which diabetes mellitus receives an insignificant share [13–15]. In addition to this, the majority of the population is socio-economically impaired and has inadequate access to healthcare facilities, which are located in the major cities [16, 17]. The costs of consultation, treatment and medicine in private sector are generally unaffordable for poor patients with diabetes mellitus [14].

An analysis of empirical literature revealed that the studies on satisfaction of diabetes patients from across the globe are rare. In earlier satisfaction studies on diabetes mellitus, the research criteria was: types of diabetes mellitus (type one and 2) [18]; mode of treatment [19, 20]; duration of illness (newly diagnosed and longtime patient) [21]; and health outcomes [22]. The present study is clinically relevant because it identifies the weaknesses and strengths of doctors that can be used to improve the process and structure of diabetes care provision. Also the tool used to conduct this study: Patient Satisfaction Questionnaire version 3 (PSQ3) is not yet tested in Pakistan.

Ware and his colleagues developed the Patient Satisfaction Questionnaire (PSQ) for measuring satisfaction of patients with four chronic diseases including diabetes [23] Scholars have used modified versions of PSQ, excluding the sub-scales inapplicable in a study context, in order to examine patient satisfaction in oncology [4]. According to Ware’s framework, the dimensions of medical interaction such as: technical expertise, interaction, communication, resources, time, convenience and availability determine patient satisfaction [24]. According to Hagedoorn and colleagues [4], PSQ3 is the most elaborate tool with basic dimensions of medical care; to measure patient satisfaction in diverse clinical settings.

Previous studies identifying specific aspects of doctor’s conduct that significantly predicted patient satisfaction showed that: taking information, listening, empathy towards patient, emotional support, friendliness, explanation of medical treatment and respect for the patient [25–27]. Furthermore, patient satisfaction is inversely related to doctors’ the use medical terms without explaining their meaning [28]. With regard to the right to respect, studies conducted in developing countries demonstrated that the patients reveal high level of acceptance for disrespect by the doctors [8]. It is perhaps a demonstration of ‘paternalistic approach’; a historical paradigm that regards doctors to be in a superior position in contrast to the patients [29]. The tolerance for disrespect and non-realization of right to respect indicate incidence of the paternalistic doctor-patient relationship in public hospitals of Pakistan [30].

Only a few authentic internationally recognised studies are available on any kind of patient satisfaction in Pakistan. The grey literature does not provide reliable information due to methodological discrepancies. To our knowledge, no study to date has specifically aimed to analyse diabetes patient satisfaction in association with dimensions of doctor-patient interaction in a free-of-cost outpatient diabetes clinic in public tertiary-care hospitals of Pakistan. In accordance with this idea, the purpose of present study were: (i) to analyse levels of patient satisfaction in association with five dimensions of doctor-patient interaction: the technical expertise, interpersonal aspects, communication, time provision and accessibility; among adult diabetes mellitus patients; (ii) to assess the factors and differences in patient satisfaction levels across patient profile characteristics; (iii) to understand the contextual particularities of satisfaction about doctor-patient interaction taking place in free of cost public diabetes clinic; in order to explain the quantified data.

## Methods

### Design

A cross-sectional mixed method triangulation design was adopted to carry out this study. The quantitative and qualitative data collection was concurrent [31, 32]. Data was collected between 22 July and 31 August 2015, in Lahore, Pakistan.

### Research setting

Lahore city was chosen to conduct this study because the highest number (eight) of public tertiary-care hospitals are located in Lahore. A list of public-sector diabetes clinics located in Lahore was obtained from the Punjab Health Department. The largest of four public diabetes outpatient clinics in Lahore, the Jinnah Allama Iqbal Institute of Diabetes and Endocrinology (JAIDE) was selected as the study setting. JAIDE provides services to approximately 28,000 diabetes outpatients every year and 150 patients every day. At the time of study, the physicians available for consultation were senior registrar, medical officers and postgraduate students in internal medicine, endocrinology, and nephrology with a sub-specialisation in diabetes mellitus. Only the serious and critical cases were referred to senior professors. The clinic was held 6 days a week, from Monday to Saturday. The schedule of on-duty doctors was flexible and patients consulted different doctors at every visit. On average two to three doctors were available in the office for consultation.

### Sampling

The representative sample was calculated using prevalence formula with: ± 3% degree of precision, 95% confidence interval, 1.96 margin of random error and an average prevalence of patient satisfaction demonstrated by three previous studies conducted in Pakistan. The sample size of 1085 diabetes patients was further adjusted for a 10% non-response rate. Thus, the total sample size before going to the field was 1164.

### Recruitment criteria

Patients for face-to-face interviews were recruited on the basis of criterion sampling method. The criteria for inclusion were: (i) adult (18 years and above), (ii) diabetes mellitus, (iii) with at least three previous visits excluding the consultation experience on the day of interview. Patients in critical condition, visiting the studied clinic inconsistently, or unwilling to participate were disqualified from the scope of this study.

### Questionnaire

The questionnaire comprised on a structured part that was based on questions from a validated tool: Patient Satisfaction Questionnaire (PSQ3). In the end of structured questionnaire, open-ended comments section was included to accommodate the qualitative information provided by the respondents. However, commenting was optional.

PSQ3 is a 50 item scale comprising favourable and unfavourable opinion statements about six dimensions of the medical care: technical expertise, interpersonal aspects, communication, time, financial costs, availability/convenience and general satisfaction [23]. Scholars have tested its applicability in diverse clinical settings, among patients with four chronic disorders, including diabetes, to measure visit-specific as well as prior experiences with inpatient, outpatient or emergency departments in public or private-sector hospitals [4, 30]. We translated the questionnaire into Pakistan’s national language, Urdu, due to concerns of comprehension, because the patients seeking healthcare at public hospitals in Pakistan are mainly poor and uneducated. The sub-scale ‘financial costs’ and six items of availability/convenience are left aside because of these are not in accordance with the research objectives of this study (Additional file 1).

We followed the specifications for translating PSQ3 provided by the Research and Development Health Corporation (RAND) [33]. The original tool was translated into Urdu language by the researchers in cooperation with an expert. The back translation of Urdu version was performed by another expert who was unacquainted with the original questionnaire. Afterwards, the questionnaire was reviewed by two experts from the Federal Bureau of Statistics, Pakistan, in order to make it culturally appropriate and comprehensible for the targeted population. This procedure was completed in multiple sessions. We ensured that the cultural applicability of concepts did not deviate from the subject matter of the original tool. The Urdu version of the questionnaire was pre-tested with 25 diabetes outpatients seeking consultations at another public diabetes clinic located in Lahore city. The final version agreed upon was used for data collection.

### Data collection

Face-to-face interviews was chosen as the medium of data collection for this survey because majority of the patients seeking consultation in JAIDE were unable to read and write. The primary author of this study collected data without any assistance. No monetary benefit was given to the respondents for participation in this study. Data was collected from 7:30 a.m. to 1:00 p.m. on consecutive days over a period of 1.5 months, 22 July to 31 August 2015. It should be noted that the clinic hours were 9 a.m. to 1 p.m. but the patients came early in the morning to for reasons beyond the scope of this study. The duration of interviews ranged from 10 to 15 min. Despite obtaining written permission for conducting this study, the access was not granted to the appointment registers as per hospital policy. Thus, the patients fulfilling eligibility criteria were approached according to the seating arrangement in associated waiting area. All the interviews were carried out after seeking verbal consent from the patients.

We developed an instruction manual for data collection on the basis of questionnaire pre-test. Debriefing sessions were held on daily basis to discuss the progress and arising problems in data collection. However, we kept records in the form of daily activity-log book and research notes. The primary author collected the data in order to maintain data integrity and avoid the chances of systematic variations in data.

With regard to the qualitative data collection, ample space was provided at the end of the structured part of the questionnaire for writing the respondent comments regarding their satisfaction about the medical encounter with doctors (optional). The comments of respondents were written down on spot, word to word by the interviewer. Sharing qualitative information was not compulsory for all recruited patients. Any patient who was willing to share qualitative information, were welcomed. Data collection continued until required sample size of 1164 was achieved. However, 1095 patients completed the structured questionnaire and 186 respondents (out of 1095 patients approached) provided qualitative information in comments section.

### Non-response and incomplete cases

Out of 1164 eligible patients approached, 3% refused to participate. Overall 1128 patients consented to participate in this study, 33 patients left the interviews incomplete. In total, 1095 respondents who completed structured part of the questionnaire were included in quantitative analysis.

### Ethical approval and quality controls

Ethical approval was successfully granted by the Ethical Review Board, University of the Punjab, before data collection. Written permission to carry out the survey was sought from the administrative head of Jinnah Hospital and Jinnah Allama Iqbal Institute of Diabetes and Endocrinology, Lahore. We made decisions regarding the methods of data collection in the design and planning phase of this study. To maintain data integrity, the researchers kept under consideration the requirements of quality assurance prior to entering the research setting and controlled the quality while data collection. Data collection quality was maintained by the use of the instruction manual with specific instructions and explanations.

The sample size was adjusted for an estimated 10% non-response rate. The actual non-response rate was 3%. The proportion of incomplete cases was around 3% of the total sample, the missing cases were excluded from the analysis [34]. The partial item missing rate of less than 5% is inconsequential [35]. In addition, we also performed pre-entry controls and used standard double data entry procedure for data management. The qualitative findings were communicated back to some of the patients recruited in this study, in order to assure trustworthiness.

### Anonymity and data protection

The interviews were conducted after seeking informed verbal consent from the patients. The respondents were provided with a brief introduction to the research objectives. In order to ensure the data privacy of respondents, all the questionnaires were assigned fictitious codes for identification. Respondents were not asked for names or identification in order to keep the information anonymous.

### Quantitative analysis

To perform statistical analysis, we computed scores for all five sub-scales: technical expertise, interpersonal aspects, communication and access/availability. There were five response categories (strongly agree, agree, uncertain, disagree, strongly disagree) for all the scale items. The response categories were recoded as: favourable statements (strongly agree = 5, agree = 4, uncertain = 3, disagree = 2, strongly disagree = 1) and unfavourable statements (strongly agree = 1, agree = 2, uncertain = 3, disagree = 4, strongly disagree = 5); so that higher scores indicate greater satisfaction. The data entry and statistical analysis was performed using SPSS (Version 22.0, IBM). The descriptive statistics were used to identify outliers and missing data. In addition to the 36 scale items of PSQ3, an overall outcome variable assessing the level of patient satisfaction with doctor-patient interaction was designed on a five-point ordinal scale from 5 (very satisfied), 4 (satisfied), 3 (undecided), 2 (dissatisfied), 1 (very dissatisfied) [2, 7]. The patients responded in three categories: very satisfied, satisfied and dissatisfied.

We performed chi-squared tests on the outcome variable and categorical social demographic variables: age, gender, education, occupation, type of residence, marital status and religion; to assess the differences across social demographics of patients and patient satisfaction. To calculate the sub-scale scores of PSQ3 among 1128 diabetes patients interviewed, the related variables were added to generate score based variables: technical expertise, interpersonal aspects, communication, time dimension and access/availability. The higher scores indicated higher satisfaction. We expressed patient satisfaction scores as mean and standard deviations. The Skewness, Kurtosis and Histograms were drawn to assess the normality of the continuous PS score variables. In addition, the Kolmogorov-Smirnov and Shapiro-Wilk tests demonstrated significant *p*-values for all continuous variables. As the data was not normally distributed, we applied Kruskal-Wallis test to assess the associations.

According to the nature of variables and study objectives, multinomial logistic regression analysis was applied. Logistic regression models were calculated to show the association between: 1) socio-demographic characteristics and patient satisfaction and 2) doctor-patient interaction and patient satisfaction. The multicollinearity was checked by drawing simple correlations between the independent variables. The goodness-of-fit test were also performed to determine if the logit model significantly predicted the outcome and fitted the data. The results are indicated by odds ratios, *p*-value (<0.05) and 95% confidence interval.

### Qualitative analysis

In order to address the third research objective of this study, we carried out a thematic content analysis of patient opinions provided in the comments section of questionnaires. It should be noted that the comments section was optional and accommodated the qualitative responses of patients recruited by criterion method. The primary researcher noted word to word the statements of patients on spot with consent of the patients, in their native languages (Urdu/Punjabi). All the questionnaires with comments were separated. The information was transcribed and translated into English prior to data analysis. The translations were checked and rechecked by the two researchers.

Using an inductive approach, dominant themes were extracted from the comments considering the re-occurring categories and irregularities [36]. Data reduction was performed manually. We classified the related comments into various categories. In this regard, the primary researcher read the interviews several times, classified the statements of patients and completed initial coding. The comments other than the scope of this study were not included in the analysis (like: cost of commuting, lack of familial support and attendants of old people etc.). In the next stage, verification of codes and generated categories was done by a second researcher. Coding sheets were prepared that summarised the generalized descriptions concerning patient perceptions along with frequencies. Finally, after adequate discussion, both researchers identified the linkages between categories. We explored the incidence of patient satisfaction/dissatisfaction along with the identification of similarities and differences among the themes, in order to grasp the subjective meanings [37]. The theme-based variables were determined by consensus.

To ensure the authenticity of qualitative findings, the first draft of findings was translated and shared with five patients individually who participated this survey and gave consent to be contacted later on. Their suggestions were obtained regarding the comprehensiveness of our findings. Except few suggestions, they agreed with the findings of this study. The changes were incorporated accordingly.

### Reflexivity

The primary author who carried out data collection and analysis, is trained in behavioural sciences and is not a medical doctor. The researcher carefully dealt with the issues of reflexivity. Records were kept in the form of memos about the challenges faced and coping strategies adopted during data collection and processing phase.

## Results

Overall, 61.4% of the diabetes mellitus patients were females and 38.6% were male. More than 20% of male patients in total sample were dissatisfied in contrast to 7.6% females. Around 57% patients were middle aged (40 to 59 years). With regard to the level of satisfaction, out of 139 (12.6%) young patients (aged: 11–39); 58% were very satisfied, 25% satisfied and 16.5% dissatisfied. The proportion of dissatisfaction was the highest among young patients as compare to the middle and old age patients. Only 9.3% of uneducated patients revealed dissatisfaction with doctor-patient interaction in contrast to 15.8% of educated counterpart. Similarly, a huge proportion of patients not doing any paid job (62.1%) revealed high satisfaction with the medical interaction (Table 1). In addition, we found a significant association between level of satisfaction and patient characteristics: gender (*X* ^2^ = 39.53, *p* < .001), education (*X* ^2^ = 12.54, *p* = .002) and occupation (*X* ^2^ = 15.50, *p* < .001).

**Table 1:** Level of satisfaction and socio-demographic characteristics of study sample (*n* = 1128)

Variables	Patient satisfaction about doctor-patient interaction in public diabetes clinic; Number (%)				
	Very Satisfied	Satisfied	Dissatisfied	Total	*p*-value^a^
Age in years					.379
11–39 40–59 60–85	81 (58.3) 375 (60) 195 (57.5)	35 (25.2) 185 (29.4) 98 (28.9)	23 (16.5) 69 (11) 46 (13.6)	139 (12.6) 629 (56.8) 339 (30.6)	
Gender					<.001
Female Male	432 (63.5) 219 (51.3)	196 (28.8) 122 (28.5)	52 (7.6) 86 (20.1)	680 (61.4) 427 (38.6)	
Education^b^					.002
Uneducated Educated	352 (62.7) 299 (54.8)	157 (28) 161 (29.5)	52 (9.3) 86 (15.8)	561 (50.7) 546 (49.3)	
Occupational status					<.001
Unemployed^c^ Employed	423 (62.1) 228 (53.5)	193 (28.3) 125 (29.3)	65 (9.6) 73 (17.2)	681 (61.5) 426 (38.5)	
Type of residence					.820
Rural Urban	150 (60.5) 501 (58.3)	68 (27.4) 250 (29.1)	30 (12.1) 108 (12.6)	248 (22.4) 859 (77.6)	
Marital status					.641
Married Not in relationship^d^	571 (59.1) 80 (56.7)	278 (28.8) 40 (28.4)	117 (12.1) 21 (14.9)	966 (87.3) 141 (12.7)	
Religion					.165
Christianity Islam	26 (74.3) 625 (58.3)	6 (17.1) 312 (29.1)	3 (8.5) 135 (12.6)	35 (3.2) 1072 (96.8)	

^a^ *p*-value based on Chi-Square test

^b^educated category is inclusive of able to read and write, few years or schooling, secondary and university level education

^c^unemployed includes: students, housewives, disabled patients

^d^separated, divorced, partner died, widow and unmarried

The mean (± SD) subscale scores of patient satisfaction were: technical expertise (38.32 ± 8.3), interpersonal aspects (23.8 ± 4.96), communication (22.38 ± 4.34), time provision (7.46 ± 2.89) and access/availability (16.17 ± 3.97). The estimates of normality demonstrated that the data was not normally distributed. We found statistically significant association of patient satisfaction with technical expertise (*X* ^2^ = 287.08, *p* < .001), interpersonal aspects (*X* ^2^ = 319.93, *p* < .001), communication (*X* ^2^ = 260.5, *p* < .001), time (*X* ^2^ = 191.13, *p* < .001) and access/availability (*X* ^2^ = 170.6, *p* < .001).

The multinomial regression analysis demonstrated that an increase in subscale scores by one unit, the likelihood of dissatisfaction decreases in contrast to being very satisfied: technical expertise (OR = .87; 95% CI = .84–.91), interpersonal aspects (OR = .82; 95% CI = .77–.87), communication (OR = .83; 95% CI = .78–.89), time dimension (OR = .90; 95% CI = .81–.99) and access/availability (OR = .78; 95% CI = .72–.84). Females are less likely than male patients to be dissatisfied than being very satisfied by the medical interaction with doctors (OR = .36; 95% CI = .23–.56). With regard to being satisfied relative to very satisfied, the likelihood of being satisfied decreases with one unit increase in subscale scores (Table 2). The likelihood ratio Chi square indicated that the model significantly predicted dependent variable (*X*2 = 68.61, *p* < .001).

**Table 2:** Factors associated with level of patient satisfaction: Results from multinomial logistic regression^a^ (*n* = 1128)

Level of satisfaction	Variables	Odds Ratio	95% CI	*p*-value
Dissatisfied	Technical expertise Time Interpersonal aspects Communication Access/availability Female	.87 .90 .82 .83 .78 .36	.84–.91 .81–.99 .77–.87 .78–.89 .72–.84 .23–.56	<.001 .043 <.001 <.001 <.001 <.001
Satisfied	Technical expertise Time Interpersonal aspects Communication Access/availability Female	.93 .93 .91 .89 .87 .91	.91–.96 .87–.99 .86–.95 .85–.94 .83–.92 .59–1.04	<.001 .017 <.001 <.001 <.001 .430

^a^Reference category: Very satisfied

### Qualitative findings

The content analysis indicated that the patient remarks were distinctively related to three categories of satisfaction: satisfactory, Undecided and dissatisfactory remarks. Patient experiences of vulnerability were linked with several aspects of doctor-patient interaction: technical expertise, time provision, alternative healthcare, approachability and communication. There were 122 female and 64 male diabetes patients who provided qualitative data. With regard to the age and education, most of the patients were illiterate (120) and aged 50 years and above (127). With regard to the health education and awareness, none of the patients reported familiarity with types of diabetes. Patients felt that their doctors never explained them about the type of diabetes they were suffering from. Amongst the four pregnant women, none was able to tell if they had gestational DM or pre-pregnancy diabetes. They told that diabetes was diagnosed during pregnancy and previously they never went to the doctor for diabetes clinical check-ups.

However, 131 patients told the mode of treatment was insulin in contrast to 55 patients used medicine. Table 3 shows frequencies of other patient characteristics including: comorbidities, present state and dependence.

**Table 3:** Profile of patients who provided qualitative data (*n* = 186)

Gender	
Male Female	64 122
Age in years	
49 and less 50 and above	59 127
Education	
Literate Illiterate	66 120
Health status^a^	
Mode of treatment Medication Insulin Comorbidities Yes No Familiarity of type of DM Patient’s current state Stable Severe Extremely critical	186 55 131 128 98 30 0 92 28 39 25
Dependence on family members	69

^a^based on subjective self-assessment and reportage of patients

### Technical expertise

The uneducated patients were unable to judge the quality of their doctors’ technical skills. Even most of the poor and uneducated patients reported inability to distinguish between a specialist and trainee doctor. Majority of the patients who responded favourably to the structured part of the questionnaire, mentioned that their satisfaction is not related to the behaviour of doctors; rather their concern is the relief from pain and solution to medical problems. This can be illustrated by the following statements of patients:

“There is nothing wrong in being snubbed by the doctors. They know better than us. So I don’t mind if my doctor is angry at me.” (Male participant)

“The problem of poor people is not respect. We do not mind if doctors insult us. All we want is for our patient to be restored to health. Our satisfaction lies in the relief from pain.” (Male participant)

*“*We are restored to health, this is what satisfies us. The rude behaviour and other things that you are asking about do not matter.” (Female participant)

Patient’s vulnerability was indicated by inability to escape from situations of disrespect, lack of alternative to public clinics, being helpless and a sense of being discriminated. In addition, the feeling of being inferior to doctors was also found to be associated with the low profile characteristics of patients. The informal conversations with the House Officers and Post Graduate trainee doctors in Medicine department of associated public hospital, highlighted that the doctors avoid taking critical cases. One of them said: “complicated cases are referred to other hospitals, if taken patients expire due to inappropriate management by service providers and insufficient diagnostic facilities. The expertise lie in saving lives and treating critical cases”. On the other hand, some patients felt that the junior doctors are doing experiments on poor patients.

**Fig. 1:**
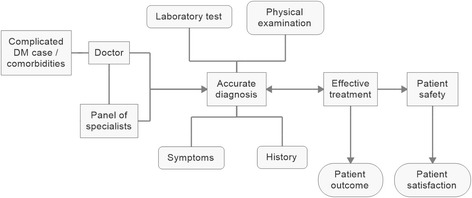
Desirability of accurate diagnosis, effective treatment and patient outcomes

**Fig. 2:**
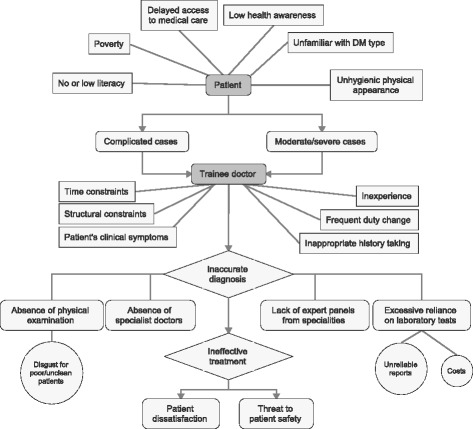
Summarizing contextual particularities in research setting

Out of 186 patients, 46 commented about various aspects of technical expertise: accuracy of diagnosis, physical examination, laboratory tests, effective treatment and experience of doctors. Almost 31 patients told that they were unable to assess technical expertise of doctors. Regarding technical shortcomings: inability of treat critical cases, inaccuracy of diagnose, excessive reliance on medical tests and an absence of physical examination of patients, the unavailability of specialist doctors for consultation, and experimentation by trainee doctors were frequently reported by the patients as the reasons of their dissatisfaction. In addition, many of patients highlighted that the doctors showed disdain towards poor patients because of their poor hygiene (Figs. 1 and 2). The question arises that if excessive reliance on medical tests does not yield accurate diagnosis, why the patients are being put under the burden of expensive laboratory tests!

### Interpersonal aspects and communication

With regard to the interpersonal and communication aspects of medical encounters, patients’ reported hesitation in asking questions from their doctors. Uneducated patients frequently mentioned that their questions and requests for repetition annoyed the physicians. Many of the middle-aged, illiterate female patients who either had low-status jobs or depended on the earnings of other family members revealed tolerance for the disrespectful conduct of doctors. They reported that they did not mind if the doctor insulted them and regarded the doctors as superior to patients. Similarly, external interruption is also tolerated (Table 4).

**Table 4:** Patterns in patient comments about the dimensions of doctor-patient interaction

Categories	Frequency of reportage by patients in categories^a^ (*n* = 186)			
	*n*	Satisfactory Remarks	Undecided/don’t know	Unsatisfactory Remarks
Technical expertise	46	5	31	10
Accuracy of diagnosis		1	4	7
Physical examination		4	3	10
Laboratory Tests		0	27	7
Effective treatment		3	2	4
Experience of doctors		1	19	9
Time dimension	38	11	–	27
Waiting time		8	–	23
Meeting interval		11	–	25
Interpersonal aspects	86	22	19	45
Friendliness		16	13	29
Feel for patient		15	9	21
Revealing disgust for unclean		–	–	39
Insulting remarks	37	22^b^	–	15^c^
Privacy		18	–	–
External interruption		13	–	5
Personal support		18	–	32
Communication	94	29	12	53
Explained about:				
Medical problem		11	9	42
Treatment plan		18	–	38
Risk factors & preventive measures		–	–	51
Information sharing				
History Taking		9	–	–
Health awareness		–	12	19
Hygiene		–	12	3
Unnecessary risk exposure		16	8	–
Answering patient’s queries		7	–	35
Listening		10	11	21
Access/availability	48	9	15	24
In-training Doctors		7^d^	10	22
Inability to consult the same doctor in repeated visits		–	–	24
Specialist doctors		9	13	–
Availability in clinic		8	15	21
Approachability of patients		6	–	21
Others	31	–	–	31
Paramedical staff behavior				26
Documentation prior to medical care				29
Availability of free medicine				31
Reliability of diagnostic facilities				24

^a^The frequency count is in accordance with the patients’ comments on more than one category of technical expertise

^b^Tolerant satisfied/Tolerant dissatisfied

^c^Intolerant satisfied/Intolerant dissatisfied

^d^Patient words: “They know that’s why sit in this large hospital”

Information sharing regarding the risks associated with a disease requires a thoughtful approach. There is difference in giving false information, unnecessary risk exposure and not sharing even the relevant risks. The information provided by the doctor regarding the risk factors affect the patient outcomes. In this study we found that doctors’ share limited information which is understandable for the uneducated patient. In doing so, important issues related to health education are ignored by the doctors like explaining the type of diabetes diagnosed and cleanliness measures for patients before seeing doctor’s clinic. Neither the doctors tell what was diagnosed nor do the patients enquire.

### Time provision

Patients told that the length of the actual consultation with doctors was less than two minutes. One of the patients said: “two minutes are even more…” Another patient: “They give us hardly two minutes… I tell you what conversation takes place when I see the doctor here… Doctor: are you taking insulin regularly? Patient: Yes. Doctor: Continue the same treatment. Patient: Doctor my sugar level is not under control. Doctor: Try to control the diet… you will be fine… next patient…“

Another important factor that affected the consultation time was the external interference. Patients told that the doctors attend phone calls, medical representatives and related staff while a patient is in the cabin. A son of a female patient in unstable condition (carried Urine bag in hand, rapid breath) told:

“When we entered the cabin two female doctors were sitting and gossiping with each other. There was no other chair on which I could make my patient sit. The doctors saw that the patient was critical but they did not bother to vacate a chair for her… she stood in great difficulty and I could not say this to the doctor. For almost ten minutes we kept standing until they asked us to tell the problem. I was just starting to tell her about my mother’s condition but a male doctor came in the cabin with a box of sweets and the both lady doctors got busy in conversation with him. I and my mother were still standing. That male doctor told them that he got engaged. The female doctors were interested in knowing how he found the girl and when he is going to get married. He told them his compete love story. When he left… we thanked God… the doctors attended us quickly and left the cabin… you can see it was still the clinic time (referring to the researcher)… the doctor did not stamp free medicine slip in hurry so we will had to request the other doctor in next cabin…”

Appropriate time is required for thorough examination and accurate diagnosis. Patients were dissatisfied with long waiting time and short meeting interval (Table 5).

**Table 5:** Synthesis of the doctor-patient interaction aspects associated with patient satisfaction/dissatisfaction (*n* = 186)

Patient experiences	Examples	Theoretical concepts
Dominance of doctor	Patients think doctor is superior to them on the basis of knowledge. Doctors can snub the patients. Patient should not counter question so the doctor may not get irritated. Patients don’t mind rudeness of doctors.	No realization of patient rights.
		Lack health education.
		Illiteracy.
		Communication is affected
External interruption	Patients are seen by doctors in presence of others unrelated people. Medical representatives come in the doctor’s room any time even when patient is being checked. Doctors attend phone calls and anyone who interrupts.	No realization of Patient right to privacy.
Time constraints	Due to dominance of doctors and short time available, the patients are unable to express their symptoms and provide history. They fear reaction of doctors.	Short meeting time.
		Long waiting time.
Inability of diagnose	Patients are not touched for physical examination. Doctors reveal disgust towards poor patients. Doctors write many irrelevant medical tests. When patients take reports to doctor, he says that these are fine now go get other tests done. After many expensive tests doctor remains unable to diagnose what exactly is the problem. In some cases, doctors recommends retests as the reports are inaccurate. Patients feel that the junior doctors are experimenting on poor people. Patients feel deprived of specialist care in public clinics. Inability to see the same doctor again.	Patients are in poor hygiene.
		Physical examination.
		Excessive reliance on tests.
		Inexperienced trainee doctors.
		Inability to judge on the basis of Clinical symptoms.
		Specialist doctors.
Unfamiliar with the type of DM diagnosed	Patients are uneducated, they cannot understand medical terminologies, cannot differentiate between specialist or trainee doctor, lack health education. They are not explained by their doctors regarding what type of diabetes they suffer from.	Vulnerability
		Lack health education
Communication with doctors	Patients feel that the doctors don’t explain in detail. They don’t appreciate asking questions. They neither convey necessary risks nor unnecessary risks to the patient.	Over/Low/Not at all informing the patient.

### Access/availability

Only a few specialist doctors are associated with the studied clinic, who are also teaching in the associated college. Not many doctors are specializing. Some are on long leave from the hospital. Hospital does not recruit new doctors. Doctors are not paid according to their services. Inability to afford consultations at private clinics leaves poor patients with public-sector healthcare facilities as their only option. This can be clearly seen in the words of one patient, who said: “We have no option except being satisfied with these doctors. We are poor… We are thankful that there is at least some place where we can seek treatment.”

## Discussion

In this paper, we examined the satisfaction of diabetes patients in association with five dimensions of doctor-patient interactions in a public diabetes outpatient clinic located in Lahore city, Pakistan. We also assessed the social-demographic variations across patient satisfaction levels. The context specific aspects influencing patient satisfaction highlighted barriers to patient safety, satisfaction and ethical health service delivery in poor resource settings. We found significant variations across levels of satisfaction: very satisfied, satisfied and dissatisfied; across gender, education and occupational status. The findings of multinomial regression analysis demonstrated that being female was associated with lower likelihood of being dissatisfied. The likelihood of dissatisfaction decreases with increasing technical expertise, interpersonal aspects, communication, time provision and access/availability. In addition, almost three-quarters of the diabetes patients who participated in this study reported higher level of satisfaction. However, contradictory remarks of diabetes patients explained quantified data.

These findings were consistent with several international studies on patient satisfaction and medical interaction [3, 6, 38–40]. The positive the patient experiences about technical expertise of doctors, the higher the level of satisfaction with medical interaction [2, 27]. In other poor resource settings where public health is free of cost; researchers have found high patient satisfaction despite disrespectful behaviour of health service providers and long waiting time [7]. Our study also highlighted that despite majority of the patients scored high satisfaction scores, there satisfaction was influenced by the absence of alternative source of consultation, tolerance of disrespect and affordability [41, 42]. Patients felt they were inferior to the doctors and were unable to judge the professional competence of doctors [43].

Almost all of the poor and uneducated respondents demonstrated an inability to understand the meaning of the latest medical developments, unnecessary exposure to risk and their right to privacy and respect. Overall, a considerable proportion of the patients responded “don’t know/uncertain” about the technical expertise of their doctors and did not realise that they had the right to be respected by their physicians [27, 44]. We believe that reducing the sense of vulnerability, enhancing a patient’s autonomy and successful treatment can lead to improvements in patient satisfaction [5]. Additionally, the patients lacked knowledge of their right to privacy and respect. A belief that doctors are superior to patients was found to be widely prevalent among uneducated and poor patients [29]. Studies showed that poverty and illiteracy are associated with higher level of satisfaction with free of cost healthcare facilities [8, 45, 46].

External interruptions and attending to calls on their mobile phones during doctor-patient meetings hindered appropriate information sharing and listening, as well as the right to privacy [32]. This explains that patients are not very much concerned with the privacy and external interruption during their medical check-up with the doctors in public sector clinics of Pakistan.

A thorough physical examination, multidisciplinary support, history and patient symptoms and laboratory tests are necessary in order to make an accurate diagnosis. In spite of relying heavily on medical tests, physicians face difficulties in making the accurate diagnosis upon which the healing of a patient depends. The patients said that doctors did not bother to touch them, which might be due to their lack of cleanliness and poor condition [9]. As our study has highlighted that the absence of physical examination is related to the poor hygiene of patients, efforts should be directed towards improving the personal hygiene of poor patients [47].

With regard to the skill of communicating with the patient is as important as the technical expertise for the doctors. In developed countries, medical graduates are trained well to share information with the patients, involve them in decisions and perform physical examination in an empathetic manner [48, 49]. Unfortunately, behavioral aspects of medical interaction are not emphasized appropriately in a scarce resource country like Pakistan. Consistent with previous studies on patient satisfaction, it is argued that the doctors should be made acquainted with the standards of ethical medical practice based on patient centered approach [50–52]. The doctors in free of cost public outpatient clinics come from economically better social strata and definitely need training to deal with poor patients. Hence, besides improving poor health awareness of patients should be made aware of their health rights: right to privacy and respect. Patients felt that an inappropriate behavior of doctors is similar to a mental torture that distresses the diabetes patients. Furthermore, patients told that they had to wait for long hours until their fasting blood sugar level is tested on the reception desk prior to see the doctor.

The lack of multidisciplinary support and inexperience of doctors was found to be associated with inaccurate diagnosis [53]. Diagnosis is the process of problem identification and depends upon thorough examination, medical tests, symptoms and history taking. In complicated cases, timely diagnosis is essential to devise an effective management plan and better chances of patient safety [48]. The barriers to accurate diagnosis, identified in this study should be addressed by the up gradation of physical and non-material culture of study setting. The quality of diagnostic services, availability of multidisciplinary care and specialist consultation are required to enhance healthcare services [54].

## Limitations and strengths

The lack of inter-hospital comparison, limited time and resources are the major limitations of this study. The hospital-based respondent selection, instead of household-based, might have missed the inclusion of dissatisfied patients who had stopped seeking medical care in public hospitals. The possibility of social desirability bias in face-to-face interviews may also limit this study. However, the large sample size, use of an internationally validated questionnaire and mixed methods triangulation are the major strengths of this study. We believe that this study provides valuable insights into the nature of doctor-patient interaction in free of cost public diabetes care clinics.

## Conclusion

The patients revealed dissatisfaction with certain aspects of the services provided by their doctors, specifically with the unavailability of specialist doctors for consultation, seeing a different doctor at every visit, inability to diagnose, the absence of a physical examination, lack of healing, treatment of complicated cases and fear of asking questions [50, 55]. The qualitative inquiry highlights the need for patient empowerment in resource-poor countries like Pakistan. A state-level strategy should be developed and implemented to control diabetes mellitus.

In order to bring services into line with the expectations of patients, the tolerance level of physicians for dealing with illiterate and poor patients should be improved, through learning expression management skills. Additionally, advanced training sequences should be organised to train doctors to deal with patients coming from the lowest strata of society. It is also suggested that the average waiting time ought to be reduced, while simultaneously increasing the duration of consultation sessions by increasing the number of specialist doctors [52]. An ethical and professional service delivery must be ensured in order to improve the quality of doctor-patient interactions in free-of-cost public outpatient diabetes care clinics in Pakistan [46, 50]. A continuous improvement in the quality of medical care service delivery can be guaranteed by conducting patient satisfaction surveys on a regular basis. State-level interventions should be directed towards strengthening public-sector health services.

## Additional file

Additional file 1: Patient satisfaction questionnaire. (DOCX 19 kb)

## Abbreviations

CI: Confidence interval
JAIDE: Jinnah Allama Iqbal Institute of Diabetes and Endocrinology
OR: Odds Ratio
PSQ: Patient Satisfaction Questionnaire
PSS: Patient Satisfaction Scores
