# Supplementary material for: Patient satisfaction with doctor-patient interactions: a mixed methods study among diabetes mellitus patients in Pakistan
Source: BMC Health Serv Res. 2017 Feb 21;17:155. doi: 10.1186/s12913-017-2094-6 (PMC5320691; doi:10.1186/s12913-017-2094-6)
Supplement: Additional file 1: — Patient satisfaction questionnaire. (DOCX 19 kb) [file 12913_2017_2094_MOESM1_ESM.docx]

**Annex II**

**Patient Satisfaction Questionnaire**

Thank you for agreeing to be a part of this research. Your opinions will be helpful in assessing the association patient satisfaction with doctors’ conduct and competence. Be assured that your responses are completely anonymous.

These are some things people say about doctors’ care giving behavior. The following questions are about how you feel about the doctors’ conduct and expertise in this institute of Diabetes. Keeping in mind your previous meetings with doctors in JAIDE, please give your responses. If you change your mind just ask the researcher to cross out your old response and make your new choice.

Anonymous Profile of Respondent

Patient serial number: _____

Age ___________ Gender ________________ Marital Status ________

Religion________________ type of place of residence (Urban/ Rural)

Permanent residence ________________________________________

Present residence ___________________________________________

Education: ______________ Occupation: ________________________

Mode of Treatment:__________________________________________

How strongly do you ‘Agree’ or ‘Disagree’ with each of the following opinion statements?

|  | | Strongly Agree | | Agree | | Uncertain | Disagree | Strongly Disagree |
| --- | --- | --- | --- | --- | --- | --- | --- | --- |
| Technical Expertise | | | | | | | | |
| 1 | Doctors are careful to check everything when treating and examining me… |  | |  | |  |  |  |
| 2 | Doctors need to be more thorough in treating and examining me… |  | |  | |  |  |  |
| 3 | I think this clinic has everything needed to provide complete medical care for diabetes |  | |  | |  |  |  |
| 4 | Sometimes, doctors make me wonder if their diagnosis is correct… |  | |  | |  |  |  |
| 5 | Doctors who treat me know about the latest medical developments…. |  | |  | |  |  |  |
| 6 | Some of the doctors I have seen lack experience with my medical problems… |  | |  | |  |  |  |
| 7 | My doctor is very competent and well-trained… |  | |  | |  |  |  |
| 8 | I have some doubts about the ability of the doctors who treated me… |  | |  | |  |  |  |
| 9 | Doctor never exposes me to unnecessary risk. |  | |  | |  |  |  |
| 10 | Doctors rarely give advice about ways to avoid illness and stay healthy. |  | |  | |  |  |  |
| Interpersonal aspects | | | | | | | | |
| 11 | Doctors act too business-like and impersonal towards me… |  | |  | |  |  |  |
| 12 | Doctors always do their best to keep me from worrying |  | |  | |  |  |  |
| 13 | While medical checkup and consultancy, doctors should pay more attention to my privacy… |  | |  | |  |  |  |
| 14 | The doctors who treat me have a genuine interest in me as a person…. |  | |  | |  |  |  |
| 15 | Sometimes, doctors made me feel foolish… |  | |  | |  |  |  |
| 16 | My doctors treat me in a very friendly and courteous manner… |  | |  | |  |  |  |
| 17 | Doctors should give me more respect… |  | |  | |  |  |  |
| Time spend with the doctor | | | | | | | | |
| 18 | Doctors usually spend plenty of time with me… |  | |  | |  |  |  |
| 19 | Sometimes, doctors hurry too much when treat me… |  | |  | |  |  |  |
| Doctor-patient Communication | | | | | | | | |
| 20 | Doctors are good about explaining the reason for medical tests. |  | |  | |  |  |  |
| 21 | Sometimes, doctors use medical terms without explaining what they mean… |  | |  | |  |  |  |
| 22 | During my medical visit I am always allowed to say everything I think is important … |  | |  | |  |  |  |
| 23 | Doctors sometimes ignore what I told them…. |  | |  | |  |  |  |
| 24 | Doctors listen carefully to what I have to say… |  | |  | |  |  |  |
| General Satisfaction | | | | | | | | |
| 25 | I am very satisfied with the medical care I receive here… |  | |  | |  |  |  |
| 26 | There are some things that could be better… |  | |  | |  |  |  |
| 27 | All things considered, medical care I receive from doctors is excellent… |  | |  | |  |  |  |
| 28 | There are some things that need to be improved… |  | |  | |  |  |  |
| 29 | Medical care I receive here is just about perfect… |  | |  | |  |  |  |
| 30 | I am dissatisfied with some things… |  | |  | |  |  |  |
| Access/ convenience and availability | | | | | | | | |
| 31 | The clinic should open for more hours |  | |  | |  |  |  |
| 32 | If I have a medical question, I can reach the doctors for help without any problem… |  | |  | |  |  |  |
| 33 | I find it hard to get an appointment right away… |  | |  | |  |  |  |
| 34 | The office hours are convenient for me… |  | |  | |  |  |  |
| 35 | I am usually kept waiting for a long time when I am at this outdoor clinic… |  | |  | |  |  |  |
| 36 | I have easy access to the medical specialist I need… |  | |  | |  |  |  |
| Overall satisfaction | | | | | | | | |
|  | | **Very satisfied** | **Satisfied** | | **undecided** | | **Dissatisfied** | **Very Dissatisfied** |
| 37 | Considering all things, I am satisfied about the medical interaction with doctors in this clinic… |  |  | |  | |  |  |

Comments of respondents:

____________________________________________________________________________________________________________________________________________________________________________________________________________________________________________________________________________________________________________________________________________________________________________________________________________________________________________________________________________________________________________________________________________________________________________________________________________________________________________________________________________________________________________________________________________________________________________________________________________
